# Situating Nutri-Ethics at the Junction of Nutrigenomics and Nutriproteomics in Postgenomics Medicine

**DOI:** 10.2174/1875692111311020008

**Published:** 2013-06

**Authors:** Nicola Luigi Bragazzi

**Affiliations:** School of Public Health, Department of Health Sciences (DISSAL), University of Genoa, Genoa, Italy

**Keywords:** Doping and personal genomics, nutri-ethics, nutrigenomics, nutriproteomics, personalized diet, personalized medicine, sports ethics.

## Abstract

Food has societal, economic, medical and ethical implications, being fundamental for life. It plays an important role also in sports medicine, since a healthy diet is an important part of an athlete's training. Nutrigenomics and nutriproteomics are emerging as a result of a convergence of nutritional, genomics and proteomics knowledge strands in the postgenomics era. These fields of inquiry present an opportunity for the design of customized diets potentially able to counterbalance the extant obesity epidemic and remedy metabolic diseases, among others. They are noteworthy for sport medicine as well since they could provide athletes with crucial information for personalized training and nutrition, in order to achieve the best results possible and express one's own potential. But they could also be used as a form of personalized doping, thus constituting an advancement of “classical nutrition-based doping” (*i.e.*, the use of nutraceuticals, stimulants and supplements). However, nutrigenomics (or nutriproteomics)-based nutritional doping is different from the first-generation doping because it is specifically tailored to the genomics and proteomics makeup of the athlete, although their effectiveness remain to be discerned in future systematic studies. Against this scientific background, ethical issues of nutrigenomics and nutriproteomics are discussed in the present paper with emphasis on the current limitations and the dizzying potentials of the omics data-intensive research for science and society. Additionally, I discuss the need to communicate uncertainty as a fundamental construct and intrinsic part of postgenomics personalized medicine, not to forget the gaps regarding the lack of adequate governance, and issues over providing a proper nutritional education to athletes as onus of the international sports organizations.

“Let food be your medicine, and medicine be your food”

Hippocrates

## 1 NEW PATHWAYS TO PERSONALIZED NUTRITION

### 1.1 Nutrigenomics and Nutriproteomics: Rise of New Postgenomics Specialties

Food, besides being a primary and basic need to sustain life, has profound societal, economic, medical and ethical implications. As a social factor, to be sure, it has shaped and deeply influenced societies, structuring their histories and ideologies and it has been used to create and shape social relationships, as well as to exert power creating unequal distribution of food resources by limiting access to food. The nutritional chain, the continuum from the production step (harvesting, manipulating, supplying, allocating and distributing) to the consumption level, has been the source of social divisions and distinctions among the social classes, between men and women, since the dawn of human history. Many social crisis and historical changes have been due to food shortages and famines, and wars have been fought for the sake of food supplies. 

As a cultural factor, food has been considered a medium of exchange, as a gift, but also as a way of defining human identity by including/excluding in one's own diet certain nutrients. Moreover, nutrition has always had an exquisite anthropological value, filling the gap between nature and culture, representing an important aspect of one's own identity and being a fascinating universe of symbols, rituals and myths [1-3].

In medicine, it is noteworthy that many diseases are in part caused by poor nutrition habits – from eating disorders to metabolic, cardiovascular pathologies and cancers and therefore nutritional education is of high importance, since according to the World Health Organization and other public health related agencies most diseases could be preventable with a proper healthy dietary lifestyle. 

Food cannot be treated using a reductionist approach; instead it is only through systems thinking that we can dissect the profound impact of nutrition on humans and population health. This is becoming particularly the case in the current postgenomics era. To this end, nutrigenomics and nutriproteomics have begun to emerge as a unique convergence of nutritional, genomics and proteomics knowledge strands since the completion of the Human Genome Project more than a decade ago. These new fields present an opportunity for the design of customized diets potentially able to counterbalance the extant obesity epidemic and remedy metabolic diseases, among others. 

Nutrigenomics (or nutritional genomics) [4] and nutriproteomics (or nutritional proteomics) [5-6] have garnered attention as distinct and highly specialized branches of postgenomics personalized medicine [7-10]. Nutrigenomics examines the food-genome intersection both in health and disease, while nutriproteomics encompasses the interactions between the nutrients and protein translation, expression and modification at a scale of the human proteome. Together, they offer the advantages of genomics such as understanding the role of hereditary factors in relation to food effects while employing proteomics so as to study gene products at the protein/proteome level. The ambitious and shared goal of nutrigenomics and nutriproteomics are to provide insights for diets that can impact gene and protein networks with a view to improving population health. They are also noteworthy for sports medicine since they could provide athletes crucial information for personalized training and nutrition, in order to achieve the best result possible and express one's own potential. The convergence of food science with omics sciences (genomics, proteomics, metabolomics or metabonomics, *etc*.) is the broader overarching tenet under which nutrigenomics and nutriproteomics are emerging, be they in drug therapy, nutritional sciences or sports medicine. 

If omics sciences call for a broader understanding of health as a complex dynamic concept situated in a social and ethical context, the application of a deterministic and reductionist approach to nascent fields of such as nutrigenomics/proteomics may lead to ethical issues and concerns. The study of ethics issues embedded in nutrigenomics and various intersections of food science with omics have been termed nutri-ethics, and discussed by various authors recently, together with analyses of responses to cope with the uncertainties of emerging postgenomics health technologies [11-16]. Nutri-ethics can be seen as an evolution of the classical concept of nutritional ethics with which it has some features in common but has also unique characteristics due to the unprecedented innovations brought along by omics disciplines.

According to one etymological analysis, the suffix “ome” present in various data-intensive omics fields is derived from the Sanskrit OM (meaning "completeness and fullness") [17, 18]. The main idea behind the data-intensive omics disciplines is that the high-throughput biomarker data obtained in parallel from successive hierarchies of cell biology can take into account the built-in molecular redundancies preserved in biology during the course of human evolution. The interactions between the human omics variation at the level of the genome, proteome, metabolome and the food are dynamic and bidirectional: in the specific cases of nutrigenomics/proteomics, they study both the network of influences of macronutrients over the human genome and the proteome and in effect, the complex responses of the human organism to food in the form of effectiveness and/or toxicity. Consequently, nutrigeno/proteomics can help modulate cellular and molecular pathways [19, 20], and foster the design and development of strategies for obesity [21], for metabolic pathologies (such as phenylketonuria) [22] or chronic diseases. Some encouraging and promising studies have shown results in the context of cancer [23, 24]. The potential of nutrigeno/proteomics is considerable [25] and includes impacts on design and development of new drugs [26, 27] but a broad consensus still lacks about safety and risk assessment using such new approaches in postgenomics medicine [28, 29].

In the case of sports medicine, nutrigeno/proteomics has been so far applied to select proper macronutrients for treating and preventing heavy exercise-induced immuno-depression, for assessing and monitoring the athlete's nutritional status and other few examples using *in vitro* and animal models [30].

Against this scientific background, ethical issues of nutrigenoproteomics are discussed in the subsequent section, with emphasis on the current limitations and the dizzying potentials of the omics data-intensive research for science and society. Additionally, I discuss the need to communicate the uncertainty as a fundamental intrinsic part of nutrigeno/proteomics, the gaps regarding the lack of adequate governance in this nascent postgenomics field, and issues over providing a proper nutritional education to the athletes as onus of the international sports organizations.

## 2 SITUATING NUTRI-ETHICS IN A POSTGENOMICS CONTEXT

It is true that nutrigeno/proteomics is a promising emerging field paving the way for personalized medicine and dietetics, even though tangible results are not likely to come along in the very near future. This calls for many ethical issues: some of these are in common with the classical ethics of nutrition or can be seen under a new light and perspective (like food safety, food medicalization, nutritional supplementation-based doping), others are absolutely novel (such as personalized nutrition, gene-nutrients interactions, personalized nutritional doping). All these issues have to be elucidated and steered with anticipatory governance and fully addressed within a coherent frame even though some aspects of my discussion concern the immediate future than the actual and urgent present [12].

Several issues, including the food safety, deserve particular emphasis. Manipulated and manufactured meals such as the engineered metabolic byproducts of essential nutrients (like beta-hydroxy-beta-methylbutyrate, or HMB, derived from leucine), novel foods like GMOs (genetically modified organisms), together with herbal preparations, phytochemical products and other kinds of enhanced / fortified meals have met with public resistance due to fears for alleged health risks, although public attitudes towards nutrigenomics/proteomics will likely vary in different global regions. Nutrigeno/proteomics could help ascertain food safety but also lead to production of functional foods, which would clearly blur the distinction between food and therapeutics. This distinction was clear for Hippocrates who said “Let food be your medicine, and medicine be your food” (see quote in the introduction), underlining the importance of a mutual relationship between these two factors, but not a priority of one over the other. In other words, seen through the lens of Hippocrates, food is for well-being but not specifically only for health. The “medicalization of food” could have negative consequences, compressing the multi-dimensionality of food values into a more narrow perspective. Food is not merely a medicine or a vehicle for drug delivery, a meal is made up of both non-functional and functional components and the act of eating has, as already stated, different functions – from building up one's own identity and sharing and communicating with others to satisfying a basic need. By blurring the boundary between food and therapeutics, all these functions could conceivably erode.

Another important concern – more technical indeed - is about the statistical reliability and robustness of the acquired nutrigeno/proteomics data that could be potentially misleading if used passively with a deterministic idea of the relationship between genes, proteins and nutrients. This is typical of nutrigeno/proteomics research, however, as the number of variables far exceeds the number of biological samples available in a given study. Uncertainty is not an accidental property of postgenomics science, but it is integral to it and must be taken into account using an anticipatory policy, as well as must be communicated as such [12].

The situation with nutrigeno/proteomics and its attendant ethical dimensions are further complicated by direct-to-consumer (DTC) tests that bypass the traditional doctors’ office; they can be ordered directly by the consumer without the involvement of a health-provider. The clinical utility of these DTC tests remain uncertain and dubious, also in part because uncertainty is often not communicated adequately. Enchanted by hype, users of DTC could utilize these test with negative impact on their health. Development of regulatory environment in the near future can help safeguard consumers’ interest, as well as educating both the health providers and DTC users [30, 31].

## 3 THE ROLE OF NUTRITION IN SPORT 

Since the time of Aristotle's “Nicomachean Ethics”, it is well known that a good, correct and balanced diet is a fundamental part of athlete’s training (the so-called “nutritional supplementation-based training”). Moreover, this diet can be differentiated according to the competing discipline, depending whether the sport is aerobic, anaerobic, which degree of power, strength, endurance is implied and so on. But even if a “sport-specific diet” exists, this is limited to some guidelines and anyway it is not tailored to the specific needs of the individual. On the other hand, it is known that the consumption of certain food and substances (like caffeine, carbohydrates) could at least in the short time modify and alter the result of a sports performance [32, 33]. However the precise effects and mechanisms of these substances are often criticized, being controversial.

The exact definition of “nutritional doping” is challenging, since it has raised a lot of doubts and objections [34]. Some scholars claim that athletes naturally use food to enhance their sports performances, differently from common reasons and motivations like suppressing cravings [34, 35]. For this reason, nutritional enhancement is just “breeding” (like Andy Miah has stated), being the specification of “functional food” superfluous for sportsmen [34, 35]. A point that should not be forgotten in the discussion is that sports training and exercises imply the production of some reactive oxygen and nitrogen species (RONS) or other oxidant molecules which lead to plasma lipids peroxidation and DNA damage at the level of muscular tissues, even though it seems that practicing regular sport would result into an adaptive response to exercise-induced oxidation [36]. However, doses of anti-oxidant supplements could restore the proper immunity system, and the market of anti-oxidant products is based on this very claiming, notwithstanding some controversial experimental findings [37]. More generally speaking, it is accepted that doing sport leads to some associated para-physiological conditions, like dehydration, fluids and ions imbalance. Bearing in mind these criticisms and considering the sports physiology as underpinned also by sports genomics, we propose to differentiate “nutritional training” - which is the use of foods normally present in diet to foster better sports performances and to restore a proper physiological status – from “nutritional doping”, which is the malicious manipulation of nutrients. Nutritional doping is when athletes use nutraceuticals, phytochemical products, megadoses of essential macronutrients, supplements and stimulants to improve and enhance their strength, or eat voluntarily meals contaminated by drugs [39, 40]. Thus they may manipulate important performance parameters and indicators, like energy supplies (by controlling the muscle contractions and energy-releasing metabolic processes), time to exhaustion and the fatigue threshold (by decreasing the production and accumulation of lactate), oxygen uptake and oxygen consumption in the muscle and other tissues, the respiratory quotient and so on. All this gives nutritionally doped athletes an unfair advantage in respect to the others, violating the fundamental principle of sports ethics. One must underline the fact that this “nutritional enhancement” does not represent the augmentation or the fulfillment of a “(genetic and biological) potential” - which should require indeed a hard work on oneself, practice, exercise, fatigue and training – but it can be seen as a short cut and as the consequence of a vision of the sport downgraded to mere business and entertainment.

So far nutritional doping is a Nobody’s land: a gray area in which there are no governing bodies and authorities that control and regulate the nutritional supplements industry. Differences in regulation between drugs and foods may lead to the false idea that nutritional supplement – and nutritional doping – may be considered legal. If the distinction between a drug and a dopant may be more clear, the barrier between food and food enhancer is blurred and undermined. Moreover, epidemiological surveys have shown that athletes have little proper and adequate knowledge about nutrition, despite the numerous sources of information being available and consult very little nutritional professionals despite their possibility of access [41].

## 4 A PERSONALIZED NUTRIGENOPROTEOMICS BASED DOPING 

Response to food and nutrients is different among the population [38], but if a personalized diet would be the ambitious goal of nutrigenoproteomics, athletes could exploit omics-based information to change consequently their diet or make use of special gene-based and engineered meals [40]. People with a certain set of alleles can metabolize some types of food in a distinctly faster fashion (including doping preparations) and so nutrigenoproteomics in sport entails with the control of both quantity and quality of food in order to achieve the desired results and possibly Olympic laurels. If a functional food being supplemented with particular nutrients and/or enriched by dopants it might not be effective in some athletes because of his/her genetic makeup, the athlete could then make use of another nutritional dopant or could modify/engineer it on the basis of omics provided information. In this sense nutritional doping has passed beyond the “one-size-fits-it-all” first generation doping in which the same dopant was used by many athletes in a not effective way to the second-generation nutritional doping with the introduction and promises of postgenomics biotechnologies. Moreover, this could make the detection of doping even more tricky and challenging, increasing the cost and the burden of anti-doping policy [42]. The peculiar aspect of this nutritional doping is that it is a *personalized* doping, since it is tailored according to the specific needs of an athlete and not just generic as the first generation or classical doping.

## 5 THE ROLE OF NUTRI-ETHICS

The role of nutri-ethics then appears central in sports ethics: due to the growing number of sports scandals, the more and more widespread and increasing doping attitude and behaviors, an ethical framework over food and nutrition, not to mention novel biotechnologies related to nutrition, are timely and crucial [43, 44]. Nutri-ethics should guide nutrigenoproteomics applications and uses, according to ethical values and ideals but prescription of these values that govern nutri-ethics in the face of nutrigenoproteomics should call for public and stakeholder deliberation. Future laboratories could help athletes in choosing healthy and doping-free foods, because some foods could be contaminated by doping agents and athletes may be not aware of this. 

Deciding to take (or not) a supplement even if legal “per se” is not an easy choice: athletes, and above all elite athletes, are constantly under pressure. For this reason, they should be supported by nutritional experts. Moreover, educational efforts should be made in advising athletes against an unrestricted and indiscriminate use of nutritional supplements. A wrong and inadequate nutrition in fact can lead to sports underperformances, due to the imbalance in nutrients concentration, in a negative energy balance, alterations of biochemical and metabolic pathways. On the other hand, nutrigenomics coupled with sports genomics may inform a better understanding of the expression of the genes related to the oxidative stress and to other paraphysiological conditions. This is of particular importance in adolescent athletes, who are particularly under pressure, being biologically and psychologically vulnerable and prone to doping use and are in a critical transition developmental phase in which metabolic, endocrine apparatus are not mature yet, as well as coping resources and resilience strategies. 

## CONCLUDING REMARKS

In this paper, I emphasized how nutrigenoproteomics is important for sport and personalized medicine since it could provide important information for personalized training, in order to achieve the best result possible and to express one's own potential since nutrition plays an important role. But nutrigenoproteomics could also be used as a platform for personalized doping, thus constituting an advancement of the “classical nutrition-based doping” (*i.e*., the use of nutraceuticals, stimulants and supplements). First-generation nutritional doping being “one-size-fits-it-all” may not be effective for all athletes and by exploiting new biomarker technologies an athlete could seek the best attainable result. The use of nutritional and nutrigenoproteomics-based doping would contravene sports ethics, being a potential enhancer and thus giving athletes advantages in the competitions not coming from their fatigue, training and motivation. Guidance from World Anti-Doping Agency (WADA) to fill in the gap by providing clear criteria to distinguish between “nutritional training” and “nutritional doping” would be useful in the age of postgenomics biotechnologies. This also calls for proper communication of the attendant uncertainty of diagnostics tests emerging in the future from nutrigenomics and nutriproteomics. 
